# Mendelian Randomization Study on hs-CRP and Dyslipidemia in Koreans: Identification of Novel SNP rs76400217

**DOI:** 10.3390/ijms26020506

**Published:** 2025-01-09

**Authors:** Ximei Huang, Youngmin Han, Minjoo Kim

**Affiliations:** 1 Department of Food and Nutrition, College of Life Science and Nano Technology, Hannam University, Daejeon 34054, Republic of Korea; 20204263@hnu.kr; 2 Institute for Health Promotion, Graduate School of Public Health, Yonsei University, Seoul 03722, Republic of Korea; popmini@naver.com

**Keywords:** dyslipidemia, high-sensitivity C-reactive protein, Mendelian randomization, causal relationship

## Abstract

High-sensitivity C-reactive protein (hs-CRP) is a marker of systemic inflammation and is associated with developing dyslipidemia. However, the causality between hs-CRP and dyslipidemia remains unresolved. This study aimed to investigate the relationship between hs-CRP concentrations and dyslipidemia and to explore the potential causal link using Mendelian randomization (MR) analysis. A nested case–control study was conducted with 1174 participants, and genotype data were analyzed using the Korean Chip. A genome-wide association study (GWAS) identified rs76400217 as a suitable instrumental variable (IV) due to its significant association with hs-CRP (*p* < 10^−8^). Logistic regression models, adjusted for confounders, were used to evaluate the association between hs-CRP and dyslipidemia. An MR analysis was performed using a two-stage least squares (2SLS) method, with rs76400217 as the IV to assess causality. Logistic regression showed a significant association between hs-CRP concentrations and dyslipidemia (OR 2.08, 95% CI: 1.81–2.39, *p* < 0.001). This association remained significant after adjusting for factors such as age, sex, alcohol consumption, and BMI. The MR analysis using rs76400217 as the IV confirmed the strong associations with hs-CRP concentrations (*p* < 0.001) in all models, but the causality between hs-CRP and dyslipidemia was not statistically significant. Thus, no evidence of a causal relationship between hs-CRP and the risk of dyslipidemia was found in the Korean population. The strong association observed between hs-CRP and dyslipidemia may be due to other contributing factors rather than a direct cause.

## 1. Introduction

Dyslipidemia is one of the most common chronic conditions, characterized by abnormal levels of serum total cholesterol (TC) and triglycerides (TG), as well as imbalances in key lipoproteins, such as elevated low-density lipoprotein cholesterol (LDL-C) and reduced high-density lipoprotein cholesterol (HDL-C) [1]. These abnormalities are widely recognized as major risk factors for atherosclerosis and cardiovascular disease (CVD) [2,3]. With the rapid modernization of Korean society and the Westernization of nutrition and lifestyle trends, such as the prevalence of high-fat, high-sugar food diets, and sedentary behaviors, the incidence of dyslipidemia has significantly increased in the Korean population [4]. According to the Dyslipidemia Fact Sheet 2022 data, in 2020, the prevalence of hypercholesterolemia among adults aged 20 years and older in Korea continued to rise, with nearly a quarter of adults being diagnosed with hypercholesterolemia and the prevalence of dyslipidemia reaching 40.2% [5]. Despite this, treatment rates remain low, raising widespread public health concerns [6].

High-sensitivity C-reactive protein (hs-CRP) is an acute-phase protein predominantly produced by the liver and mature adipocytes, serving as a marker for tissue damage, infection, and inflammation [7]. Recognized as a classical biomarker of chronic low-grade inflammation, hs-CRP is crucial in evaluating the risk of various diseases, including CVD [8], cancer [9], and type 2 diabetes mellitus (T2DM) [10]. Epidemiological evidence indicates that individuals with dyslipidemia frequently exhibit a pro-inflammatory state, characterized by elevated concentrations of inflammatory cytokines such as tumor necrosis factor-α (TNF-α), interleukin-1β (IL-1β), and interleukin-6 (IL-6), which further stimulate hs-CRP production [11,12]. A study focusing on a Korean cohort identified a correlation between dyslipidemia and increased hs-CRP concentrations [13], highlighting the potential role of inflammation in the pathogenesis of dyslipidemia. Nonetheless, interpreting these associations in observational studies is complicated by the presence of confounding factors and reverse causality, making it difficult to ascertain whether there is direct causation between these variables [14,15]. In particular, while there is a known association between hs-CRP and dyslipidemia, this relationship may be confounded by shared factors such as lifestyle or environmental influences, or it may be a consequence of the disease itself affecting hs-CRP concentrations. Thus, further research is needed to better understand their relationship and its potential implications for clinical decision-making.

The Mendelian randomization (MR) approach has emerged as a valuable method to address these limitations. MR utilizes genetic variations as instrumental variables (IVs) for exposure factors, as these genetic variations are less prone to confounding by environmental or lifestyle factors [16,17]. By simulating the structure of randomized controlled trials (RCTs), the MR approach helps to mitigate confounding and reverse causation issues inherent in traditional observational studies [18]. Numerous studies have employed MR to investigate causality between modifiable exposures and major non-communicable diseases, including coronary artery disease (CAD) [19], T2DM [20], and cancer [21]. However, the causality between hs-CRP and dyslipidemia has not yet been explored using MR. Thus, this study aims to investigate the causality between hs-CRP concentrations and dyslipidemia through MR analysis, offering new insights into the underlying mechanisms.

## 2. Results

### 2.1. Demographic and Clinical Characteristics According to the Presence of Dyslipidemia

This study included 1174 participants, of whom 581 had normal lipid concentrations and 593 had been diagnosed with dyslipidemia. A comparison of the demographic and clinical characteristics between the two groups is shown in Table 1. The results indicated that the average age in the dyslipidemia group was significantly higher than that in the normal group (*p* < 0.001), and the proportion of males was lower (*p* = 0.016). In terms of lifestyle factors, the proportion of alcohol drinkers was significantly lower in the dyslipidemia group (*p* = 0.001), but there was no significant difference in the proportion of smokers. The prevalence of prediabetes or T2DM was significantly higher in the dyslipidemia group compared to the normal group (*p* = 0.002).

Anthropometric measurements showed that although the waist circumference in the dyslipidemia group was slightly smaller than in the normal group, this difference was not statistically significant. However, the body mass index (BMI) in the dyslipidemia group was significantly higher than that in the normal group (*p* = 0.032). Additionally, the two groups had no significant differences in systolic and diastolic blood pressure (BP).

In terms of metabolic indicators, insulin levels, homeostasis model assessment of insulin resistance (HOMA-IR), and hemoglobin A1c (HbA1c) were all significantly higher in the dyslipidemia group compared to the normal group (*p* < 0.05). A comparison of lipid profiles and inflammatory markers showed that TG, TC, LDL-C, hs-CRP, oxidized low-density lipoprotein (ox-LDL), and 8-epi-prostaglandin F_2α_ (8-epi-PGF_2α_) were all significantly higher in the dyslipidemia group, while HDL-C was slightly lower (*p* = 0.033). After adjusting for confounders such as sex, age, BMI, alcohol consumption, and prediabetes or T2DM, the variables that remained statistically significant included TG, TC, LDL-C, hs-CRP, MDA, ox-LDL, and 8-epi-PGF_2α_ (Table 1).

### 2.2. Genotype Distribution

In this study, the distribution of rs76400217 C>T genotypes among all participants was as follows: 1 individual was homozygous for the T allele (TT), 84 individuals were heterozygous (TC), and the remaining 1089 individuals were homozygous for the C allele (CC). The genotype frequencies did not significantly deviate from the Hardy–Weinberg equilibrium (*p* > 0.05). We combined the heterozygous (TC) and rare homozygous (TT) groups for analysis to enhance statistical power.

### 2.3. Association of rs76400217 with Clinical and Metabolic Parameters

**Figure 1:**
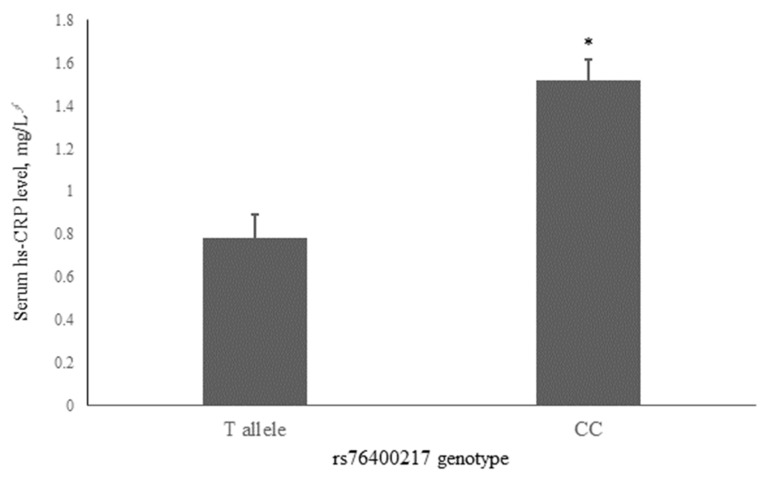
Associations of serum hs-CRP with the rs76400217 C>T. Mean ± standard error (SE) concentrations of hs-CRP for the T allele and CC genotypes are shown by gray bars. * p < 0.001 was derived using an independent *t*-test. *^∮^* Tested following logarithmic transformation. hs-CRP, high-sensitivity C-reactive protein.

To account for potential confounding, we investigated the association of rs76400217 with common risk factors for dyslipidemia, including the presence of dyslipidemia (Table 2). The results showed that individuals with the CC genotype had the highest mean hs-CRP concentrations (1.52 ± 0.10 mg/dL, *n* = 1089), while carriers of the T allele had lower hs-CRP concentrations (0.78 ± 0.11 mg/dL, *n* = 85) (*p* < 0.001, Figure 1). However, as shown in Table 2, there were no significant differences in the prevalence of prediabetes or T2DM, or dyslipidemia between genotype groups. Additionally, no significant differences were found in other lifestyle factors, clinical characteristics, and metabolic parameters, including sex, alcohol consumption, smoking status, age, waist circumference, BMI, BP, glucose levels, lipid profiles, and oxidative stress markers.

### 2.4. Association Between the hs-CRP and Dyslipidemia

A two-stage least squares (2SLS) analysis assessed the causality between hs-CRP and dyslipidemia (Table 3). The results indicated that each additional risk allele of rs76400217 was significantly associated with changes in hs-CRP concentrations across all models (*p* < 0.001), with F-statistics ranging from 16 to 33, suggesting that rs76400217 is an effective IV for hs-CRP. However, despite the effectiveness of rs76400217 as an IV, the association between hs-CRP concentrations and dyslipidemia did not reach statistical significance in any of the models, with odds ratios (ORs) of 1.44 (*p* = 0.267), 1.50 (*p* = 0.240), 1.51 (*p* = 0.228), 1.48 (*p* = 0.277), 1.49 (*p* = 0.264), and 1.52 (*p* = 0.236).

The logistic regression models examined the association between hs-CRP concentrations and dyslipidemia after adjusting for various confounding factors. In the crude model (Model 1), the OR was 2.08 (95% confidence interval [CI]: 1.81–2.39; *p* < 0.001), indicating a strong association. After adjusting for age and sex (Model 2), the OR slightly increased to 2.11 (95% CI: 1.83–2.43), and the association remained highly significant (*p* < 0.001). Further adjustments for alcohol consumption (Model 3), BMI (Model 4), and both alcohol consumption and BMI (Model 5) produced similar results, with ORs ranging from 2.10 to 2.11 and all *p*-values remaining below 0.001. In Model 6, which additionally accounted for prediabetes or T2DM, the OR remained stable at 2.09 (95% CI: 1.81–2.43; *p* < 0.001).

## 3. Discussion

This research used MR to examine whether there is a causal link between hs-CRP and dyslipidemia. Through a genome-wide association study (GWAS), the SNP rs76400217 was found to be significantly associated with hs-CRP concentrations. By using this SNP as an IV, we aimed to minimize confounding effects from environmental and lifestyle factors. Although observational studies suggested a positive correlation between hs-CRP and dyslipidemia, the MR analysis did not support the direct causation between elevated hs-CRP and dyslipidemia.

The results of this study align with previous research [13], which identified a significant association between elevated hs-CRP concentrations and dyslipidemia. However, the relationship between hs-CRP and dyslipidemia remains unclear. Several cross-sectional studies have shown a positive correlation between hs-CRP and lipid levels, such as TC, TG, and LDL-C, while showing a negative correlation with HDL-C [22,23,24]. In contrast, other studies reported associations in the opposite direction [25] or found no correlation at all. These inconsistencies may arise due to differences in study populations, including variations in ethnicity, lifestyle, and genetic background. Given the broad application of hs-CRP as an inflammatory marker and its ease of measurement [26], further research is necessary to better understand its role in managing dyslipidemia risk.

Despite the observational evidence linking hs-CRP to dyslipidemia, the MR analysis did not confirm a direct causal effect. This outcome is consistent with MR studies in other disease contexts, where observational studies have shown significant associations between CRP and diseases like cancer, CVD, and non-alcoholic fatty liver disease, but the MR analysis failed to establish direct relationships [27,28,29]. Moreover, MR studies on CRP and hypercholesterolemia have yielded varied results across different populations. Si et al. [30] reported that genetically determined CRP concentrations were positively associated with hypercholesterolemia in the FinnGen population, while no significant effect was observed in the UK Biobank cohort. These discrepancies suggest that while CRP is associated with various diseases as a marker of inflammation, it may not be a direct driver of pathogenic mechanisms. A more comprehensive understanding of the complex interactions between inflammation and lipid metabolism is still needed, as highlighted in recent reviews of meta-analyses of observational studies [31]. As the first study to use MR to analyze the causality between hs-CRP and dyslipidemia, this research indicates that confounding factors may influence the association or that a direct causal link might not exist, with other underlying mechanisms playing a role instead. Future research should further explore this area to better understand the intricate interactions between inflammation and lipid metabolism.

The results of this study suggest that hs-CRP may function more as a risk marker for dyslipidemia rather than as a causative factor. This finding enhances our understanding of the biological interactions between hs-CRP and lipid metabolism. Both hs-CRP and dyslipidemia are well-established biomarkers for CVD and atherosclerosis [32,33], and research increasingly indicates that lipid levels and inflammation influence each other, contributing to disease progression. Lipids, such as cholesterol and saturated fats, can trigger inflammatory responses, while inflammation-related proteins like hs-CRP can affect lipid metabolism and arterial health [34]. Further investigations into these relationships could help clarify the role of these biomarkers in CVD risk assessment and inform future intervention strategies.

Notably, this study identified a novel association between rs76400217 and hs-CRP concentrations in a Korean population. This discovery is particularly significant, as no prior research has examined this SNP. Rs76400217, located in the intronic region of the LOC105370802 gene [35], has been linked to various phenotypes, including idiopathic scoliosis and Parkinson’s disease [36]. Additionally, LOC105370802 has been identified as one of 16 critical genes in lung adenocarcinoma prognosis studies, highlighting its potential role in cancer progression [37]. Research by Lee et al. [38] has shown that another SNP within LOC105370802, rs951295, is significantly associated with changes in DNA methylation levels, particularly under the influence of smoking and cadmium exposure, emphasizing its relevance in environment-related diseases. Identifying the association between rs76400217 and hs-CRP levels adds to our understanding of the role of LOC105370802 in inflammatory responses and opens up new avenues for future research. Further studies should explore the phenotypic associations of this SNP across diverse populations and investigate its potential links with other inflammatory markers, such as IL-6 and TNF-α. If subsequent research confirms the role of rs76400217 in inflammation and metabolic diseases, this SNP could become a novel biomarker for assessing disease risk or treatment response, offering significant clinical utility.

This study does have some limitations. The sample is predominantly from the Korean population, which may limit the applicability of the findings to other ethnic groups or regions. Additionally, while MR analysis helps control for confounding variables, it cannot fully eliminate the possibility of unmeasured confounding factors. Furthermore, the use of the National Cholesterol Education Program Adult Treatment Panel III (NCEP-ATP III) guidelines from 2002 for the diagnosis of dyslipidemia may not reflect the lower thresholds recommended by more recent guidelines. While the NCEP-ATP III criteria are widely recognized and allow for comparability with previous studies, future research could consider adopting updated diagnostic standards to align with contemporary clinical practice and validate these findings in a broader context. Despite these limitations, this study is the first to report a significant association between rs76400217 and hs-CRP concentrations in the scientific literature. It is also pioneering in its investigation of the potential causality between the inflammatory marker hs-CRP and dyslipidemia. Moreover, this study minimized biases related to reverse causation and confounding by selecting a representative general population sample, excluding individuals with pre-existing conditions, and controlling for various key clinical factors. Together, these findings provide valuable insights into the complex biological pathways connecting inflammation and lipid metabolism.

Another critical point is that while this study is limited to the Korean population, it opens the door for future research to investigate the potential role of rs76400217 in other inflammation-related diseases. The identification of such genetic markers could be crucial in developing personalized therapeutic strategies for managing dyslipidemia and related conditions.

## 4. Materials and Methods

### 4.1. Study Population

The sample size was determined based on the estimated effect sizes from previous studies examining similar associations, along with the desired statistical power (80%) and significance level (*p* = 0.05). To mitigate the risk of potential dropouts and missing data, a larger initial sample was recruited than required. In this nested case–control study, 1404 participants were initially assessed for eligibility. These individuals were recruited between 2014 and 2019 from the National Leading Research Laboratory of Clinical Nutrigenetics/Nutrigenomics at Yonsei University and the National Health Insurance Corporation of Ilsan Hospital in Goyang, Republic of Korea. All participants were informed about the study’s objectives and provided written informed consent. The study protocol was approved by the Institutional Review Board of Hannam University (2023-04-08-0405) and adhered to the principles of the Declaration of Helsinki.

**Figure 2:**
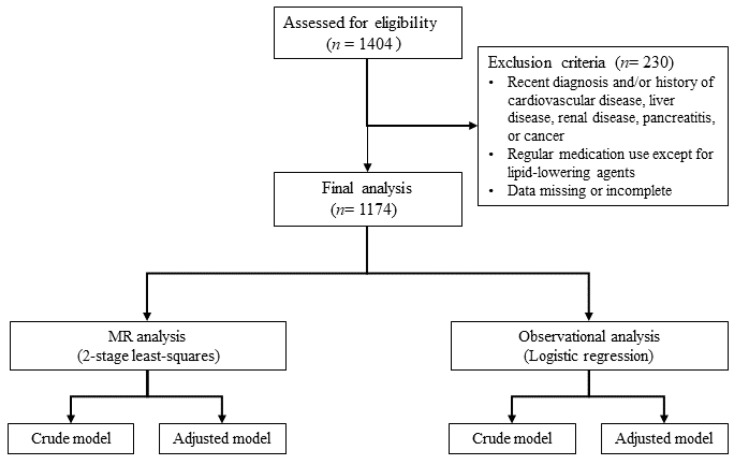
Flow chart of study. MR, Mendelian randomization.

After applying exclusion criteria, which included recent diagnoses or histories of cardiovascular disease, liver disease, renal disease, pancreatitis, or cancer, regular use of medications (excluding lipid-lowering agents), and accounting for missing or incomplete data, 230 participants were excluded. The diseases were identified based on self-reports from the participants during their routine medical check-ups, using standardized hospital-provided questionnaires. As a result, 1174 participants, aged 22 to 86, were included in the final analysis. The detailed selection process is depicted in Figure 2.

### 4.2. Definition of Disease

Dyslipidemia in this study was defined under the guidelines of the NCEP-ATP III [39]. Participants were classified as having dyslipidemia if they met at least one of the following criteria: TG concentrations of 150 mg/dL or higher, LDL-C concentrations of 130 mg/dL or higher, HDL-C concentrations below 40 mg/dL, or TC concentrations of 200 mg/dL or higher. The diagnosis of prediabetes and T2DM was based on the criteria defined by the American Diabetes Association, which includes fasting serum glucose levels of ≥126 mg/dL for T2DM and 100–125 mg/dL for prediabetes, or HbA1c levels of ≥6.5% for T2DM and 5.7–6.4% for prediabetes [40].

### 4.3. Lifestyle and Anthropometric Assessments

Participants completed a standardized questionnaire to assess lifestyle factors. Smoking status was categorized into three groups: never-smoker, ex-smoker, and current smoker. Alcohol consumption was classified into two categories: nondrinkers and current drinkers.

Anthropometric measurements, including weight, height, waist circumference, and BP, were collected following standardized procedures. BMI was calculated from the measured weight and height as weight (kg) divided by height squared (m^2^). Waist circumference was recorded at the umbilical level following normal expiration while the participant stood. BP was measured with an automatic monitor (FT-200S; Jawon Medical, Gyeongsan, Republic of Korea) after a minimum rest period of 20 min.

### 4.4. Biochemical Assessments

Venous blood and urine samples were collected after a 12 h fast and then stored at −80 °C and −20 °C, respectively. Serum lipid levels, including TG, TC, and HDL-C, were measured using enzyme kits (Daiichi, Tokyo, Japan). LDL-C was calculated using the Friedewald formula. Fasting glucose and insulin concentrations were determined with commercial kits (Siemens, Tarrytown, NY, USA; DIAsource, Louvain la-Neuve, Belgium), and insulin resistance (IR) was calculated using HOMA-IR: [fasting insulin (μIU/mL) × fasting glucose (mmol/L)]/22.5. Additional measurements included HbA1c by immunoturbidimetric analysis, hs-CRP with a hs-CRP-Latex kit (DenkaSeiken, Tokyo, Japan), plasma malondialdehyde (MDA) using a TBARS assay kit (ZeptoMetrix Co., Buffalo, NY, USA), urinary 8-epi-PGF_2α_ with an ELISA kit (Oxford Biomedical Research Inc., Rochester Hills, MI, USA), and ox-LDL with an enzyme immunoassay (Mercodia AB, Uppsala, Sweden). Measurements were performed with a Wallac 1420 Victor multilabel counter (PerkinElmer Life Sciences, Boston, MA, USA).

### 4.5. SNP Genotyping and Selection

Genotyping was carried out with the Affymetrix Axiom™ KORV1.1-96 Array and Axiom^®^ 2.0 Reagent Kit (Affymetrix, Santa Clara, CA, USA) following the manufacturer’s protocol. Detailed procedures are described in a previous publication [13]. Genotype data were generated with the Korean Chip (K-CHIP).

To identify SNPs significantly associated with hs-CRP, a GWAS was conducted. Among the top ten SNPs associated considerably with hs-CRP, only rs76400217 met the stringent significance threshold (*p* < 10^−8^). Consequently, rs76400217 was determined to be a suitable instrumental variable for this analysis and was included in subsequent analyses to explore its role further.

### 4.6. Statistical Analysis

All statistical analyses were conducted using IBM SPSS Statistics 26.0 and RStudio v2024.04.2 software. To address the skewed distributions observed in glucose markers, lipid profiles, and oxidative stress markers, logarithmic transformations were applied. This approach normalized the data distributions, minimized the influence of outliers, and ensured the validity of statistical analyses by aligning with the assumptions of parametric tests. Independent *t*-tests were performed to compare continuous variables between control and dyslipidemia groups, as well as among different genotypes. Chi-square tests were used for categorical variables to assess frequency distributions. The UNIANOVA statistical method was employed to adjust for confounding variables. Logistic regression models were employed to calculate the OR with 95% CI for the association between hs-CRP and dyslipidemia. Two-tailed *p*-values < 0.05 were considered statistically significant. GWAS analysis was carried out using PLINK 1.9, with linear regression analyses used to evaluate the association between genotypes and hs-CRP concentrations.

The MR analysis was conducted using a 2SLS regression approach to explore the causality between hs-CRP and dyslipidemia. In the first stage, hs-CRP was regressed using the IV. The second stage involved regressing dyslipidemia using the predicted values from the first stage.

## 5. Conclusions

This study is the first to identify a significant association between the SNP rs76400217 and hs-CRP. However, the MR analysis did not provide evidence for a direct causal relationship between hs-CRP and dyslipidemia risk. These findings suggest that while hs-CRP may serve as a marker of inflammation, it is unlikely to be a direct cause of dyslipidemia. This research underscores the potential role of rs76400217 as an important genetic marker in inflammation and encourages further exploration into the biological mechanisms linking hs-CRP and lipid metabolism. Additional studies are required to fully understand how hs-CRP influences the development of dyslipidemia and how inflammation contributes to metabolic diseases.

## Figures and Tables

**Table 1:** Comparison of characteristics in normal and dyslipidemia individuals.

	Normal (*n* = 581)	Dyslipidemia (*n* = 593)	*p*	*p* ^a^	*p* ^b^	*p* ^c^
Male sex (*n*, %)	223 (38.4)	188 (34.7)	0.016	-	-	-
Alcohol drinker (*n*, %)	376 (64.7)	329 (55.5)	0.001	0.069	-	-
Current smoker (*n*, %)	85 (14.6)	82 (13.8)	0.366	0.367	0.267	0.233
Prediabetes or T2DM (*n*, %)	191 (32.9)	248 (41.8)	0.002	0.014	0.012	-
Age (year)	48.0 ± 0.47	50.5 ± 0.43	<0.001	-	-	-
Waist (cm)	82.7 ± 0.31	82.0 ± 0.30	0.138	0.121	0.108	-
BMI (kg/m^2^)	23.6 ± 0.11	23.9 ± 0.10	0.032	0.014	0.016	-
Systolic BP (mmHg)	118.7 ± 0.63	119.9 ± 0.60	0.169	0.337	0.345	0.877
Diastolic BP (mmHg)	73.7 ± 0.47	74.7 ± 0.40	0.081	0.190	0.172	0.476
Glucose (mg/dL) ^∮^	95.3 ± 0.72	97.0 ± 0.95	0.258	0.430	0.385	0.372
Insulin (μIU/dL) ^∮^	8.99 ± 0.24	9.21 ± 0.17	0.034	0.028	0.034	0.140
HOMA-IR ^∮^	2.15 ± 0.08	2.20 ± 0.05	0.018	0.022	0.025	0.274
HbA1c (%) ^∮^	6.02 ± 0.05	6.26 ± 0.07	0.006	0.001	0.001	0.053
Free fatty acids (μEq/L) ^∮^	554.6 ± 11.0	583.2 ± 10.7	0.013	0.102	0.073	0.076
Triglycerides (mg/dL) ^∮^	80.8 ± 1.23	127.6 ± 3.23	<0.001	<0.001	<0.001	<0.001
Total-cholesterol (mg/dL) ^∮^	172.0 ± 0.77	203.2 ± 1.38	<0.001	<0.001	<0.001	<0.001
HDL-cholesterol (mg/dL) ^∮^	56.4 ± 0.46	55.8 ± 0.61	0.033	0.011	0.019	0.069
LDL-cholesterol (mg/dL) ^∮^	99.4 ± 0.76	121.9 ± 1.31	<0.001	<0.001	<0.001	<0.001
hs-CRP (mg/L) ^∮^	1.40 ± 0.14	1.53 ± 0.11	<0.001	<0.001	<0.001	<0.001
Malondialdehyde (nmol/mL) ^∮^	8.81 ± 0.14	9.75 ± 0.18	<0.001	<0.001	<0.001	<0.001
Ox-LDL (U/L) ^∮^	35.7 ± 0.73	41.3 ± 0.91	<0.001	0.002	0.001	0.007
8-epi-PGF_2α_ (pg/mg creatinine) ^∮^	1072.2 ± 36.0	1638.8 ± 29.9	<0.001	<0.001	<0.001	<0.001

Data are presented as frequencies (%) and mean ± standard error (SE). *p*-values were derived using an independent *t*-test for continuous variables and a Chi-square (χ^2^) test for categorical variables. *p* ^a^-values were adjusted for sex and age. *p* ^b^-values were adjusted for sex, age, and drinking. *p* ^c^-values were adjusted for sex, age, BMI, drinking, and prediabetes or T2DM. ^∮^ Tested by logarithmic transformation. T2DM, type 2 diabetes mellitus; BMI, body mass index; BP, blood pressure; HOMA-IR, homeostasis model assessment of insulin resistance; HbA1c, hemoglobin A1c; HDL-cholesterol, high-density lipoprotein cholesterol; LDL-cholesterol, low-density lipoprotein cholesterol; hs-CRP, high-sensitivity C-reactive protein; Ox-LDL, oxidized low-density lipoprotein; 8-epi-PGF_2α_, 8-epi-prostaglandin F_2α_.

**Table 2:** Characteristics of individuals according to the rs76400217 C>T.

	T Allele (*n* = 85)	CC (*n* = 1089)	*p*
Male sex (*n*, %)	24 (28.2)	387 (35.5)	0.174
Alcohol drinker (*n*, %)	47 (55.3)	658 (60.4)	0.353
Current smoker (*n*, %)	12 (14.1)	155 (14.2)	0.551
Prediabetes or T2DM (*n*, %)	34 (40.0)	405 (37.2)	0.606
Dyslipidemia (*n*, %)	38 (44.7)	555 (51.0)	0.267
Age (year)	48.8 ± 1.24	49.3 ± 0.33	0.695
Waist (cm)	80.9 ± 0.92	82.5 ± 0.22	0.052
BMI (kg/m^2^)	23.3 ± 0.28	23.7 ± 0.08	0.140
Systolic BP (mmHg)	118.1 ± 1.63	119.4 ± 0.45	0.451
Diastolic BP (mmHg)	73.7 ± 1.11	74.3 ± 0.32	0.625
Glucose (mg/dL) ^∮^	95.9 ± 2.37	96.1 ± 0.62	0.765
Insulin (μIU/dL) ^∮^	9.90 ± 1.00	9.04 ± 0.13	0.953
HOMA-IR ^∮^	2.35 ± 0.27	2.16 ± 0.04	0.963
HbA1c (%) ^∮^	6.39 ± 0.17	6.14 ± 0.05	0.129
Free fatty acids (μEq/L) ^∮^	563.0 ± 30.8	569.4 ± 7.94	0.484
Triglycerides (mg/dL) ^∮^	94.2 ± 6.48	105.3 ± 1.95	0.062
Total-cholesterol (mg/dL) ^∮^	185.7 ± 3.19	187.9 ± 0.96	0.591
HDL-cholesterol (mg/dL) ^∮^	57.7 ± 1.60	56.0 ± 0.40	0.330
LDL-cholesterol (mg/dL) ^∮^	109.2 ± 2.86	110.9 ± 0.86	0.642
Malondialdehyde (nmol/mL) ^∮^	9.65 ± 0.52	9.26 ± 0.12	0.593
Ox-LDL (U/L) ^∮^	38.2 ± 1.91	38.7 ± 0.62	0.234
8-epi-PGF_2α_ (pg/mg creatinine) ^∮^	1430.1 ± 91.5	1352.8 ± 25.7	0.658

Data are presented as frequencies (%) and mean ± standard error (SE). *p*-values were derived using an independent *t*-test for continuous variables and a Chi-square (χ^2^) test for categorical variables. ^∮^ Tested by logarithmic transformation. T2DM, type 2 diabetes mellitus; BMI, body mass index; BP, blood pressure; HOMA-IR, homeostasis model assessment of insulin resistance; HbA1c, hemoglobin A1c; HDL-cholesterol, high-density lipoprotein cholesterol; LDL-cholesterol, low-density lipoprotein cholesterol; hs-CRP, high-sensitivity C-reactive protein; Ox-LDL, oxidized low-density lipoprotein; 8-epi-PGF_2α_, 8-epi-prostaglandin F_2α_.

**Table 3:** Association of hs-CRP with dyslipidemia risk using MR analysis and logistic regression.

	Change in hs-CRP per Risk Allele				MR Analysis		Logistic Regression	
	β	SE	F	*p*	OR (95% CI)	*p*	OR (95% CI)	*p*
Model 1	0.688	0.120	32.7	<0.001	1.44 (0.76–2.75)	0.267	2.08 (1.81–2.39)	<0.001
Model 2	0.667	0.119	19.4	<0.001	1.50 (0.76–2.93)	0.240	2.11 (1.83–2.43)	<0.001
Model 3	0.672	0.119	16.3	<0.001	1.51 (0.77–2.94)	0.228	2.10 (1.82–2.42)	<0.001
Model 4	0.635	0.117	28.5	<0.001	1.48 (0.73–3.00)	0.277	2.11 (1.82–2.44)	<0.001
Model 5	0.640	0.117	24.1	<0.001	1.49 (0.74–3.01)	0.264	2.10 (1.82–2.44)	<0.001
Model 6	0.644	0.117	20.3	<0.001	1.52 (0.76–3.08)	0.236	2.09 (1.81–2.43)	<0.001

Model 1: crude model. Model 2: adjusted for age and sex. Model 3: adjusted for age, sex, and drinking. Model 4: adjusted for age, sex, and BMI. Model 5: adjusted for age, sex, BMI, and drinking. Model 6: adjusted for age, sex, BMI, drinking, and prediabetes or T2DM. hs-CRP, high-sensitivity C-reactive protein; MR, Mendelian randomization; SE, standard error; OR, odds ratio; CI, confidence interval.

## Data Availability

The data presented in this study are available on request from the corresponding author. The data are not publicly available due to privacy or ethical restrictions.
